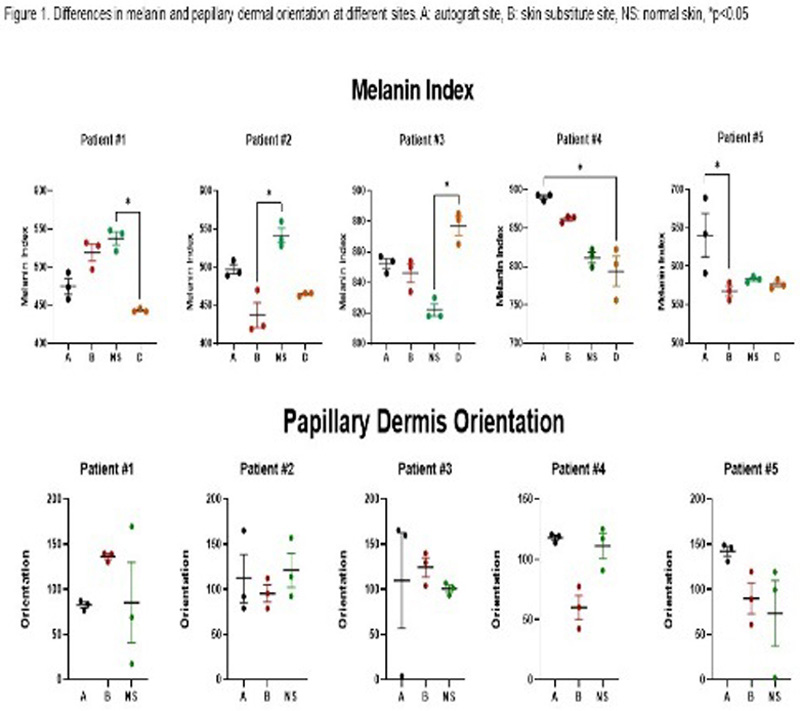# 517 Long Term Scarring Assessment of Burn Wounds Treated with Bioengineered Allogeneic Cellularized Construct

**DOI:** 10.1093/jbcr/irad045.114

**Published:** 2023-05-15

**Authors:** Eriks Ziedins, Alina Corona, Melissa McLawhorn, Sarah Burkey, Cara Delatore, Alison Ross, Jeffrey Shupp, Bonnie Carney

**Affiliations:** Firefighters' Burn and Surgical Research Laboratory, Washington, DC; Firefighters' Burn and Surgical Research Laboratory, Washington, DC; Firefighters' Burn and Surgical Research Laboratory, MedStar Washington Hospital Center, Washington, DC; MHRI, Frederick, Maryland; Firefighters Burn and Surgical Research Lab, Washington, DC; Firefighters' Burn and Surgical Research Laboratory, MedStar Health Research Institute, Washington, DC, Washington, DC; Firefighters' Burn and Surgical Research Laboratory, MedStar Health Research Institute, Georgetown University School of Medicine, Washington, DC; Firefighters' Burn and Surgical Research Laboratory, Medstar Health Research Institute, Georgetown University Medical Center, Washington, DC; Firefighters' Burn and Surgical Research Laboratory, Washington, DC; Firefighters' Burn and Surgical Research Laboratory, Washington, DC; Firefighters' Burn and Surgical Research Laboratory, MedStar Washington Hospital Center, Washington, DC; MHRI, Frederick, Maryland; Firefighters Burn and Surgical Research Lab, Washington, DC; Firefighters' Burn and Surgical Research Laboratory, MedStar Health Research Institute, Washington, DC, Washington, DC; Firefighters' Burn and Surgical Research Laboratory, MedStar Health Research Institute, Georgetown University School of Medicine, Washington, DC; Firefighters' Burn and Surgical Research Laboratory, Medstar Health Research Institute, Georgetown University Medical Center, Washington, DC; Firefighters' Burn and Surgical Research Laboratory, Washington, DC; Firefighters' Burn and Surgical Research Laboratory, Washington, DC; Firefighters' Burn and Surgical Research Laboratory, MedStar Washington Hospital Center, Washington, DC; MHRI, Frederick, Maryland; Firefighters Burn and Surgical Research Lab, Washington, DC; Firefighters' Burn and Surgical Research Laboratory, MedStar Health Research Institute, Washington, DC, Washington, DC; Firefighters' Burn and Surgical Research Laboratory, MedStar Health Research Institute, Georgetown University School of Medicine, Washington, DC; Firefighters' Burn and Surgical Research Laboratory, Medstar Health Research Institute, Georgetown University Medical Center, Washington, DC

## Abstract

**Introduction:**

Bioengineered allogeneic cellularized construct (BACC) has recently been proven to be effective for treatment of burn wounds and now has FDA approval. There is a paucity of data describing long term (≥1 year) scar characteristics following treatment with BACC, including its potential to modulate the formation of hypertrophic scar (HTS). HTS causes significant physical and psychosocial implications which can result in diminished quality of life. This study aims to characterize post-burn scars resulting from BACC-treated wounds compared to within subject matched autografted wounds using clinical and histologic metrics.

**Methods:**

Adult patients (n=5) were previously enrolled in a Phase III clinical trial for treatment of their deep partial thickness acute burn wounds. Subjects had clinical scar examinations including imaging, Scar Assessment Scales (POSAS), and non-invasive skin measurements to quantify skin elasticity, erythema, and melanin. Punch biopsies were collected and fixed in formalin from the autograft-treated site (A), the BACC-treated site (B), the donor-site (D), and an area of normal skin (NS). The formalin-fixed biopsies were then embedded in paraffin, sectioned, and stained with H&E, Picrosirius Red, and Herovici.

**Results:**

The use of BACC produced no difference in POSAS score and achieved elasticity measurements comparable to NS (Table 1). The erythema measurement demonstrated differences between scar sites and each scar site was different from NS. The donorsite also had some variation from NS. The measured melanin levels were different between each scar site, and both were different from NS. The donorsite also had detectable differences in melanin from NS. Measurements of epidermal thickness demonstrated differences between scar sites with BACC being similar to NS. Rete ridge ratio were different between scar sites and between autograft vs. NS. Papillary dermis cellularity had minor differences between scar sites and both scar sites were different from NS. Reticular dermis cellularity was equivalent between autograft and BACC sites and both were different from NS. Papillary and reticular alignment were not different between scar sites. Reticular orientation was not different between autograft and BACC but was different in BACC vs. NS. Papillary dermal thickness was different between autograft and BACC.

**Conclusions:**

The HTSs resulting from wound closure achieved using BACC are similar to autografting in metrics related to clinical POSAS scores, extracellular matrix, and collagen structure. Scars following autografting or the use of BACC to achieve wound closure differ in metrics related to pigmentation, epidermal structure, and papillary dermal thickness and cellularity.

**Applicability of Research to Practice:**

These findings may suggest that the use of BACC is a more optimal treatment for certain burn depths in certain patients because some HTS outcomes are equivalent, and it eliminates the need for a donor site.